# Phakomatosis Pigmentovascularis Associated With Sturge–Weber Syndrome, Ota Nevus, and Congenital Glaucoma

**DOI:** 10.1097/MD.0000000000001025

**Published:** 2015-07-02

**Authors:** Yangfan Yang, Xiujuan Guo, Jiangang Xu, Yiming Ye, Xiaoan Liu, Minbin Yu

**Affiliations:** From the State Key Laboratory of Ophthalmology, Zhongshan Ophthalmic Center, Sun Yat-sen University, Guangzhou, China.

## Abstract

Phakomatosis pigmentovascularis (PPV) is a rare congenital malformation syndrome that is characterized by a combination of capillary abnormalities and dermal melanocytosis.

We describe 3 cases of PPV combined with bilateral Sturge–Weber syndrome (SWS), Ota nevus, and congenital glaucoma.

Case 1 was a 2-year-old boy. Facial port-wine stains distributed along the 3 branches of his trigeminal nerves, which suggested the existence of SWS. Gray-blue patches were spread over the frontal and temporal areas of bilateral face, waist, buttocks, and thigh. Bilateral triangular alopecia was found on the temporal scalp. The diagnosis of Ota nevus was made by the bilateral scleral malanocystosis. Increased intraocular pressure, enlarged cornea, and pathologic optic disc cupping supported the diagnoses of infantile bilateral glaucoma. Case 2 was a 4-year-old boy. Port-wine stains were found on the face along the 3 branches of the trigeminal nerve and distributed along the trunk, arms, and legs. Mongolian spots spread over his frontal and temporal areas of the bilateral face, waist, buttocks, thigh, abdomen, and back. Infantile glaucoma was found in both eyes. Ota nevus were found in the both eyes. Optic coherent tomography (OCT) scans revealed increased thickness of choroid. Case 3 was a 5-year-old boy. Besides Ota nevus and infantile glaucoma in both eyes, color Doppler ultrasonography showed choroidal hemagioma. OCT scan showed increased choroidal thickness. The bilateral triangular alopecia on the child's temporal scalp was similar to that of Case 1. Cases 1 and 2 presented with port-wine stain patches that were consistent with the characteristic manifestation of PPV type IIb. However, the CMTC of Case 3 met the diagnostic criteria for PPV type Vb.

Case 1 was treated with trabeculotomies in both eyes. For Cases 2 and 3, surgical interventions were not considered due to the high risks of antiglaucomatous operation complications. We prescribed them antiglaucoma indications.

The simultaneously coexistence of PPV with SWS, Ota nevus, and congenital glaucoma is rare. In the clinic, additional detailed examinations and tests of PPV patients to exclude other ocular abnormalities or extraocular involvements are necessary.

## INTRODUCTION

Phakomatosis pigmentovascularis (PPV) is a rare congenital malformation syndrome that is characterized by a combination of capillary abnormalities and dermal melanocytosis that are present from birth and have been classified by Hasefawa and Yasuhara into 4 types according to the different characteristics of the vascular and pigmentary malformations.^1^ Each type is further subdivided into 2 subtypes based on the absence (type a) or presence of extracutaneous involvement (type b). In 2003, Torrelo et al described PPV type V with cutismarmorata telangiectatica congenita (CMTC) associated with aberrant Mongolian spots.^2^ Recently, a new classification of PPV was proposed by Happle, who rejected PPV type I and redefined types II, III, IV, V as cesioflammea, spilorosea, cesiomarmorata, and unclassifiable, respectively (Table 1).^3^ Approximately 50% of PPV patients have associated and 1 or more comorbidities, including Sturge–Weber syndrome (SWS), Ota nevus, Klippel–Trenaunay syndrome, congenital glaucoma, mental retardation, hydrocephalus, congenital triangular alopecia (CTA), and skeletal abnormalities.^4^

**TABLE 1:**
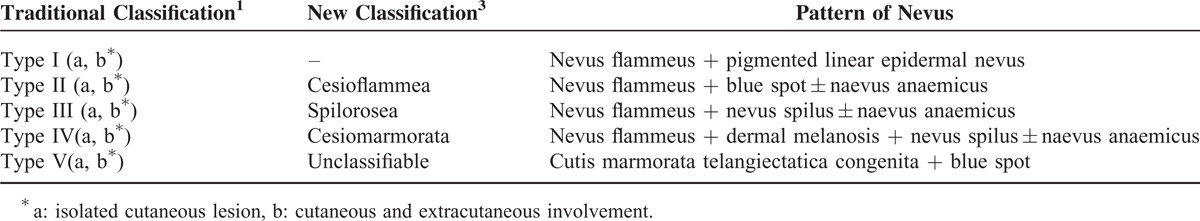
Classification of Phacomatosis Pigmentovascularis

Here, we report 3 patients with similar clinical manifestations that were characterized by cutaneous vascular and pigmentary malformations that were complicated with bilateral Sturge–Weber syndrome, Ota nevus, and congenital glaucoma. The most distinguished differences between these 3 cases involved the features of the cutaneous erythemas. Two patients presented with port-wine stain patches that were consistent with the characteristic manifestation of PPV type IIb. The cutaneous lesions of the other case were characterized by reticular, marble-like lesions (CMTC) that met the diagnostic criteria for PPV type Vb.

## METHODS

A retrospective review of 3 consecutive patients with phakomatosis pigmentovascularis and associated bilateral SWS, Ota nevus, and congenital glaucoma who were evaluated at Zhongshan Ophthalmic Center was conducted. Ethics approval was obtained from the Human Ethics Committee of Zhongshan Ophthalmic Center (No. 2014MEKY026). All clinical examinations and treatment adhered to the tenets of the Declaration of Helsinki. Parents of all 3 patients gave written informed consent.

All patients received complete ophthalmic evaluations that included assessments of intraocular pressure (IOP), anterior segment and fundus examinations, gonioscopy with a RetCam and ocular ultrasonography. The patients also underwent optic coherent tomography (OCT) to determine the thicknesses of the retinal nerve fiber layer and the choroid.

Full blood tests, serum chemistries, electrocardiograms, chest X-rays, and brain magnetic resonance imaging (MRI) were also conducted to rule out possible systemic abnormalities.

## RESULTS

Only 3 patients with PPV combined with SWS, Ota nevus, and congenital glaucoma were found in the Zhongshan Ophthalmic Center (a major tertiary subspecialty center in Guangzhou, China) over 10 years. Two patients were referred for examination and treatment for suspected glaucoma from dermatologic departments, and 1 patient was referred for high IOPs (>40 mm Hg) in both eyes that were identified in a routine health checkup organized by a kindergarten. The 3 patients were Chinese boys ranging in age from 2 to 5 years. The family histories were unremarkable. Their patients were nonconsanguineous couples.

## CASE REPORTS

### Case 1: 2-Year-Old Male

A 2-year-old Chinese boy was referred to Zhongshan Ophthalmic Center (a major tertiary subspecialty center in Guangzhou, China) for examination and treatment for suspected glaucoma. He was the second son of a nonconsanguineous couple, whose elderly son was not found to have any congenital abnormality. The family history was unremarkable.

### Systemic Clinical Findings

**FIGURE 1:**
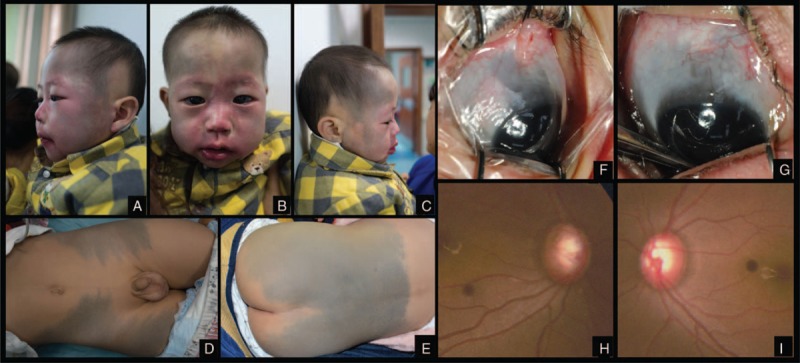
Case 1. Facial port-wine stains distributed along the branches of trigeminal nerve. Gray-blue patches (Mongolian spot) spread over forehead, temporal. Triangular alopecia existed symmetrically on bilateral temporal scalp (A, B, C). Gray-blue patches (Mongolian spot) spread over thigh (D). Gray-blue patches (Mongolian spot) spread over waist and breech (E). Scleral malanocystosis on the right eye (F). Scleral malanocystosis on the left eye (G). Enlarged optic disc cup on the right eye (H). Enlarged optic disc cup on the left eye (I).

General examination revealed that facial port-wine stains distributed along the 3 branches of the trigeminal nerve, which was consistent with the cutaneous manifestation of Sturge–Weber syndrome (Figure 1A–C). Meanwhile, the boy was also found greyish blue patches (Mongolian spots) spreading over the frontal and temporal areas of bilateral face, waist, buttocks, and thigh (Figure 1D, E). All of the skin lesions were presented since birth. Apart from the vascular and pigmentary lesions, the child also presented with triangular alopecia which was symmetrical on the temporal side bilaterally (Figure 1A, C). No mental retardation or abnormal physical development has been found in neurological examinations of the central nervous system by a neuropaediatrician.

### Ocular Clinical Findings

The lesions on the eyeballs were characterized by bilateral scleral malanocystosis, suggesting the diagnosis of Ota nevus (Figure 1F, G). The IOP was 25.8 mm Hg in the right eye and 21.8 mm Hg in the left. Cornea was clear and diameter was 12 mm in both eyes. Cup-to-disc area ratio was 0.7 in the right eye and 0.5 in the left eye (Figure 1H, I). Ophthalmic examinations above suggested infantile bilateral glaucoma with increased IOP, enlarged cornea, and pathologic optic disc cupping.

### Image Findings and Systemic Test

OCT scans were unremarkable. Brain MRI scan was normal. No abnormality was found in full blood test, serum chemistry, electrocardiogram, and chest X-ray.

### Treatment Selection

Two weeks after confirming the diagnosis of congenital glaucoma, the boy was treated by trabeculotomy in both eyes. Intraocular pressure (IOP) at 3 months after operation was 12.6 mm Hg in the right eye and 13.3 mm Hg in the left eye.

### Case 2: 4-Year-Old Male

A 4-year-old Chinese boy was referred to Zhongshan Ophthalmic Center for examination and treatment for suspected glaucoma. He was the only son of a nonconsanguineous couple. The family history was unremarkable.

### Systemic Clinical Findings

**FIGURE 2:**
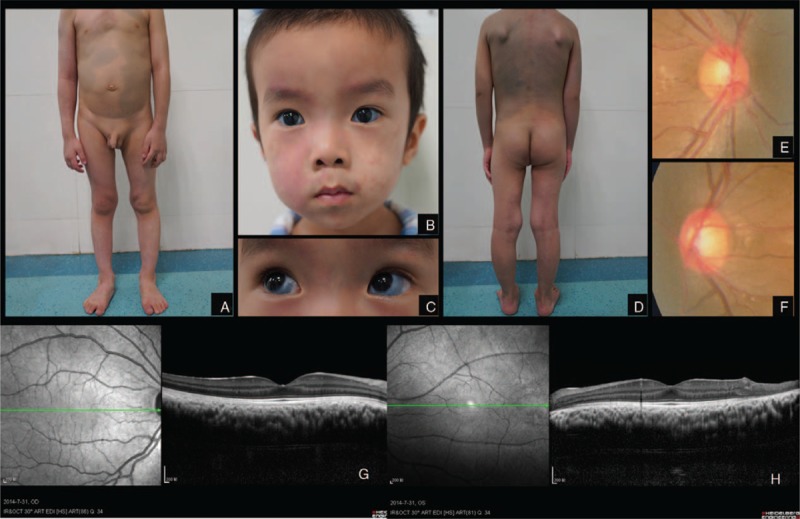
Case 2. Port-wine stains distributed along chest, arms, and legs. Greyish blue patches (Mongolian spots) spreading over abdomen (A). Port-wine stains were found on the face along the 3 branches of trigeminal nerve (B). Bilateral scleral malanocystosis (C). Port-wine stains distributed along back, arms, and legs. Mongolian spots spreading over back (D). Enlarged optic disc cup on the right eye (E). Enlarged optic disc cup on the left eye (F). Increased choroidal thickness in the right eye on OCT scan (G). Increased choroidal thickness in the left eye on OCT scan (H).

Port-wine stains were found on the face along the 3 branches of the trigeminal nerve (Figure 2B) and distributed along the trunk, arms, and legs (Figure 2A, D). Greyish-blue patches (Mongolian spots) spreading over the frontal and temporal areas of the bilateral face, waist, buttocks, thigh, abdomen, and back (Figure 2A, D).

### Ocular Clinical Findings

The IOP was 25 mm Hg in the right eye and 27 mm Hg in the left eye. Cornea was clear and diameter was 13 mm diameter in the right eye and 12.5 mm in the left eye. Cup-to-disc area ratio was 0.5 in right eye and 0.6 in left eye (Figure 2E, F). Bilateral scleral malanocystosis was found on the eyeballs, suggesting the diagnosis of Ota nevus (Figure 2C).

### Image Findings and Systemic Test

OCT scans revealed increased thickness of choroid (Figure 2G, H). The parents of this patient refused to let their child undergo color Doppler ultrasonography or any further test. Therefore, choroidal hemangioma was just suspected in this case.

Brain MRI scan was normal. No abnormalities were found in the results of full blood tests, serum chemistries, electrocardiograms, or chest X-rays.

### Treatment Selection

For this case, surgical interventions were not considered due to the high risks of antiglaucomatous operation complications, such as fulminant hemorrhage in the epichoroidal space due to choroidal hemangioma. We prescribed hypotensive agents and regular follow-up visits for him. His IOP was normal (16.7 mm Hg in the right eye,10.0 mm Hg in the left eye under 2 eye drops at the latest follow-up). No changes of optic cup/disc ratio in both eyes were observed.

### Case 3: 5-Year-Old Male

A 5-year-old boy was referred to Zhongshan Ophthalmic Center (Guangzhou, China) for high IOP (>40 mm Hg) in both eyes which was found in a routine health checkup organized by the kindergarten. Before the referral, the boy was diagnosed as bilateral congenital glaucoma and treated using 3 kinds of hypotensive eye drops. He was the only son of a nonconsanguineous marriage. No family history was remarkable.

### Systemic Clinical Findings

**FIGURE 3:**
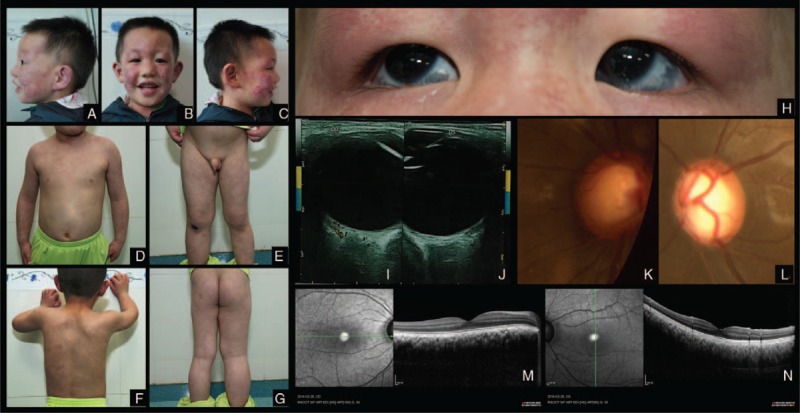
Case 3. Reticular, marble-like vascular lesions (CMTC) on the face along the branches of trigeminal nerve. Bilateral triangular alopecia on temporal scalp (A–C). CMTC on the neck, chest, arms and hands, aberrant blue spots on the chest and abdomen (D). CMTC and aberrant blue spots on the back (F). CMTC on the legs. Hemihypertropy on the right lower extrimety (E, G). Scleral hyperpigmentation in both eyes (H). Increased choroidal thickness and increased internal blood flow in the lesion on color Doppler ultrasonography in both eyes (I, J). Enlarged optic cup in both eyes (K,L). Increased choroidal thickness in the right eye on OCT scan (M). Increased choroidal thickness in the left eye on OCT scan (N).

Reticular, marble-like lesions (cutismarmorata telangiectatica congenita (CMTC)) were found on the face along the 3 branches of trigeminal nerve of both sides, neck, bilateral chest, buttocks, arms, hands, and legs (Figure 3A–G). The boy had multiple aberrant Mongolian blue spots on the abdomen and back (Figure 3D, F). His lower right extremity showed soft tissue hypertropy (Figure 3E, G). The bilateral triangular alopecia on the child's temporal scalp was similar to that of Case 1 (Figure 3A, C).

### Ocular Clinical Findings

Ophthalmologic examinations revealed scleral hyperpigmentation, suggesting Ota nevus (Figure 3H), enlarged cornea, and optic disc cupping in both eyes (Figure 3K, L). Cornea was clear and diameter was 13 mm Hg in both eyes. The IOP was 20 mm Hg in the right eye and 15 mm Hg in the left eye.

### Image Findings and Systemic Test

Color Doppler ultrasonography showed increased internal blood flow in the choroid, which suggests choroidal hemagioma (Figure 3K, L). Optic coherent tomography (OCT) scan showed increased choroidal thickness (Figure 3M, N).

Brain MRI scan was normal. The results of blood routine test, serum chemistry, electrocardiogram, and breast and bilateral lower extremity X-ray were negative.

### Treatment Selection

Considering high risks of antiglaucomatous operation complications, surgical intervention was not considered and the child had been on 3 topical medications. Follow-up examinations were carried out on a regular basis. The IOP in his latest eye check (his third visit, 3 months from the first) was 18 mm Hg in the right eye and 19 mm Hg in the left eye. No changes of optic cup/disc ratio in both eyes were observed.

## DISCUSSION

The term phakomatosis pigmentovascularis describes a congenital coexistence of cutaneous lesions with vascular and pigmentary sources. Since its first definition by Ota et al in 1947, more than 200 cases of PPV have been reported worldwide.^5^ The pathogenesis is hypothesized to result from dysplasia of the vasomotor nerve cells and melanin cells of the embryonic neural crest.^6^ The “twin-spotting” phenomenon or the loss of genetic heterozygosity is thought to be responsible for the simultaneous occurrence of 2 different congenital abnormalities in PPV.^3,7,8^ Vascular and melanotic genetic mutations occur in each of a pair of homologous chromosomes; subsequently, stem cells from the mutant pair generate mixed homologous daughter cells from 2 different mutations.^8^

A common extracutaneous involvement in PPV is SWS, which is classically characterized by facial angioma in the divisions of the trigeminal nerve and unilateral or bilateral occipital leptomeningeal angiomata. Facial angiomas distributed in ophthalmic division (V1) are strongly but not completely predictive of glaucoma and/or leptomeningeal angiomata.^9^ According to the Roach Scale, SWS is classified into the following 3 types: type I (facial and leptomeningeal angiomas ± glaucoma (classic SWS); type II (facial angioma alone ± glaucoma), and type III (leptomeningeal angioma).^10^ In our cases, the facial angiomas were distributed along all 3 divisions of the trigeminal nerve, were combined with bilateral glaucoma, and exhibited no involvement of the central nervous system; thus, we considered these cases to be type II.

PPV complicated with congenital glaucoma might not solely occur due to SWS. The abnormal pigmentation at the anterior angle caused by ocular hyperpigmentation (Ota nevus) that mechanically blocks the outflow of aqueous humor might be another mechanism.^11^ Teekhasaenee et al suggested that extensive angioma and melanocytosis involving the globe is associated with a strong predisposition for congenital glaucoma.^12^

PPV combined with bilateral SWS, Ota nevus, or congenital glaucoma are uncommon conditions. Future molecular and genetic studies will reveal the exact mechanism of the association between PPV and these 3 comorbidities.

In conclusion, we reported 3 cases of PPV associated with SWS, bilateral glaucoma, and congenital triangular alopecia. One case was PPV type IIb, and the other case was PPV type Vb. In ophthalmic clinics, glaucoma with dermal angiomas and melanocytosis should be considered to be PPV, and intensive examinations and tests to exclude other ocular abnormalities (eg, choroidal angioma and choroidal melanoma) and extraocular involvement (eg, leptomeningeal angiomas) are warranted to develop an ocular treatment plan and systemic therapy.

## CONCLUSION

The simultaneous coexistence of PPV with SWS, Ota nevus, and congenital glaucoma is rare. In the clinic, additional detailed examinations and tests of PPV patients to exclude other ocular abnormalities or extraocular involvements are necessary.
